# Recurrent HSV-2 Meningitis: A Case of Mollaret’s Meningitis Misdiagnosed for Years

**DOI:** 10.7759/cureus.91154

**Published:** 2025-08-28

**Authors:** Ali Haider, Hassan Rizwan, Fayyas Ahamed, Hasanur Rahman, Mohamed Abdulmajeed

**Affiliations:** 1 General Internal Medicine, Luton and Dunstable University Hospital, Luton, GBR; 2 Medicine, Divisional Headquarters Teaching Hospital Mirpur, Azad Jammu and Kashmir, PAK; 3 Internal Medicine, Luton and Dunstable University Hospital, Luton, GBR; 4 General Internal Medicine, Luton and Dunstable University Hospital, Luton , GBR

**Keywords:** herpes simplex virus, hsv meningitis, infectious disease, mollaret's meningitis, viral meningitis

## Abstract

We present a rare case of Mollaret’s meningitis in a middle-aged man who experienced recurrent episodes of aseptic meningitis over several years, repeatedly misdiagnosed and treated empirically without definitive virological confirmation. Diagnosis was eventually established by detecting herpes simplex virus type 2 (HSV-2) DNA in cerebrospinal fluid (CSF) via polymerase chain reaction (PCR). Awareness of this condition and prompt PCR testing facilitated appropriate antiviral therapy and avoidance of unnecessary broad-spectrum antibiotics. This case highlights the importance of considering HSV-2 in patients with recurrent lymphocytic meningitis and educating them about the benign, recurrent nature of this syndrome.

## Introduction

Mollaret’s meningitis, also known as benign recurrent lymphocytic meningitis, is an uncommon neurological syndrome characterized by repeated episodes of aseptic meningitis interspersed with symptom-free intervals. First described by Pierre Mollaret in 1944, the syndrome eluded precise etiology for decades until the advent of molecular diagnostics. Subsequent research established a strong association with herpes simplex virus type 2 (HSV-2), which is now recognized as the primary causative agent in the majority of cases [1].

Patients typically present with sudden-onset headache, fever, photophobia, and nuchal rigidity, mimicking acute bacterial meningitis but without the associated mortality or neurological sequelae. These episodes are generally self-limiting, resolving within days to weeks without long-term damage. However, recurrent episodes can lead to significant psychological distress, unnecessary hospitalizations, and inappropriate antimicrobial use [2,3]. The rarity and under-recognition of this condition frequently result in delayed or missed diagnoses, highlighting the need for heightened clinical awareness.

Molecular confirmation of HSV-2 DNA in cerebrospinal fluid (CSF) via polymerase chain reaction (PCR) has emerged as the gold standard diagnostic modality, replacing older cytologic methods [4]. CSF cytology may still play a supportive role, particularly in settings lacking rapid PCR availability. Other herpesviruses such as HSV-1 and Epstein-Barr virus have been implicated in rare cases, but HSV-2 accounts for the majority [5,6].

Epidemiologically, Mollaret’s meningitis most frequently affects young to middle-aged adults, with a slight female predominance. Clinical cohorts have reported lymphocytic pleocytosis, normal glucose, and elevated protein in CSF as consistent findings during active episodes [7,8]. While the condition is typically benign, the relapsing course can severely impact patients' mental health and quality of life, contributing to a cycle of emergency presentations, extensive workups, and unwarranted treatments. This case report presents a patient with a history of recurrent aseptic meningitis who was ultimately diagnosed with HSV-2-related Mollaret’s meningitis, highlighting the role of PCR diagnostics, antiviral therapy, and patient counseling in effective disease management.

## Case presentation

**Figure 1:**
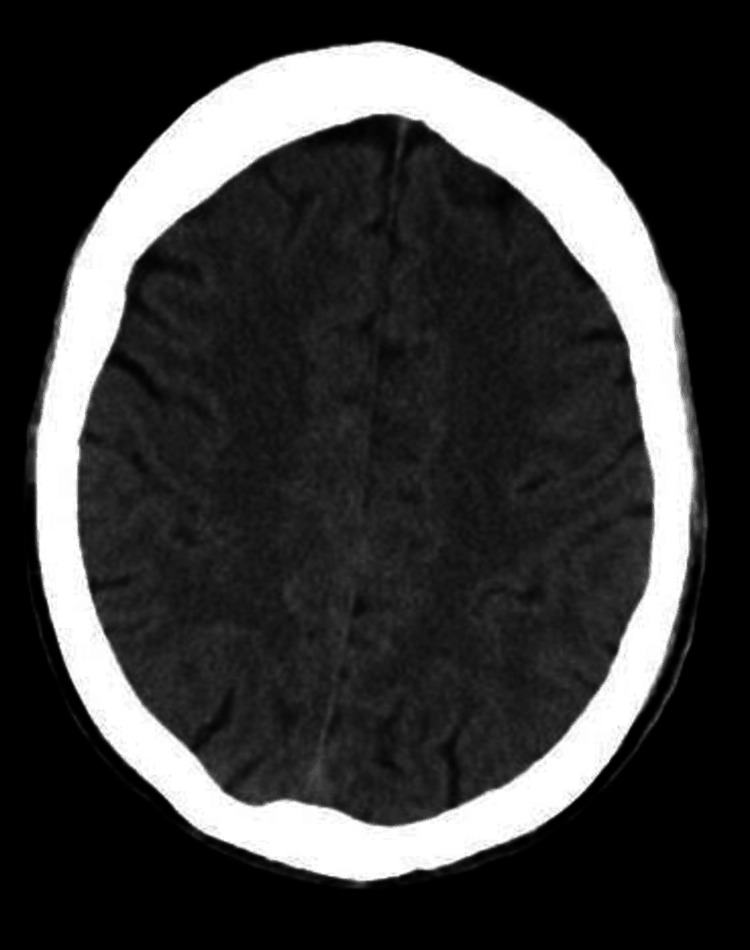
Normal CT head

A 57-year-old male presented to the emergency department with a three-day history of severe left-sided headache, facial pain, photophobia, neck stiffness, fever, nausea, vomiting, and dizziness. His medical history included multiple prior hospital admissions over several years for similar meningitic symptoms, previously diagnosed as “presumed viral meningitis” and treated empirically with intravenous (IV) acyclovir and broad-spectrum antibiotics. Other notable history included depression, anxiety, and recurrent cystitis. The patient reported that during previous episodes, symptoms would resolve spontaneously after hospitalization, but he experienced considerable anxiety about the recurrent nature of his condition. On examination, he appeared unwell, febrile (38.5°C), and exhibited neck stiffness and photophobia but no focal neurological deficits. There were no skin lesions or genital ulcers. Neurological examination was otherwise normal. In the acute setting, the patient was initially started empirically on IV acyclovir (10 mg/kg every eight hours) and IV chloramphenicol (25 mg/kg every six hours), the latter selected due to his documented severe allergy to both penicillins and cephalosporins, pending results of cultures and PCR testing. A CT head was done and was normal (Figure 1). MRI of the brain was not performed during this admission, as the patient remained neurologically stable with no focal deficits and CSF PCR confirmed HSV-2 infection, making further neuroimaging unnecessary at that stage. Dexamethasone was administered as a single dose to address severe meningeal inflammation while awaiting diagnostic confirmation. Once CSF PCR confirmed HSV-2 (Table 1) and bacterial cultures remained negative, the antibiotics were discontinued after consultation with microbiology. He was transitioned to oral valacyclovir 1000 mg three times daily for 14 days.

**Table 1:** Laboratory investigations CSF: cerebrospinal fluid; PCR: polymerase chain reaction; WBC: white blood cell count; CRP: C-reactive protein; mm³: cubic millimeters; mmol/L: millimoles per liter; g/L: grams per liter; mmH₂O: millimeters of water

Test	Patient’s result	Reference range
White blood cell count (WBC)	9.2 ×10⁹/L	4–11 ×10⁹/L
Neutrophils	6.1 ×10⁹/L	2–7.5 ×10⁹/L
Lymphocytes	2.0 ×10⁹/L	1–4 ×10⁹/L
C-reactive protein (CRP)	12 mg/L	<5 mg/L
Serum glucose	5.6 mmol/L	3.5–6.0 mmol/L
CSF opening pressure	220 mmH₂O	100–250 mmH₂O
CSF WBC	180/mm³ (lymphocyte predominant)	<5/mm³
CSF protein	0.65 g/L	0.15–0.45 g/L
CSF glucose	3.4 mmol/L	>2.5 mmol/L
Blood cultures	No growth at 36 h	—
CSF bacterial culture	No growth	—
CSF PCR	Positive for HSV-2	Negative

To address the psychological impact of his recurrent illness, the patient was offered a consultation with the hospital’s liaison psychiatry team. In addition, he was counseled extensively on the benign nature of his condition, and the option of initiating long-term suppressive therapy with valacyclovir 500 mg once daily was discussed. The patient declined immediate suppressive therapy but agreed to close outpatient follow-up with neurology, where this option could be reconsidered if recurrences persisted. Preventive measures, including avoidance of known triggers such as stress and fatigue, were emphasized.

The patient was started empirically on IV acyclovir and IV chloramphenicol upon admission while awaiting results of cultures and PCR. Once CSF PCR confirmed HSV-2 and bacterial cultures remained negative, the antibiotic therapy was discontinued after consultation with microbiology. He was switched to oral valacyclovir 1000 mg three times daily for 14 days based on national guidelines [5]. He improved clinically, with resolution of fever and gradual relief of headache over the next few days. He was discharged with outpatient neurology follow-up and education regarding the recurrent but benign nature of Mollaret’s meningitis. Long-term suppressive antiviral therapy was discussed but deferred at this stage.

## Discussion

Mollaret’s meningitis is a rare syndrome of recurrent aseptic meningitis typically due to HSV-2 [1]. The condition often goes unrecognized for years, as in this case, where repeated episodes were treated empirically without definitive diagnosis.

The clinical presentation usually involves sudden onset of headache, fever, meningismus, and occasionally cranial nerve palsies or altered sensorium [2]. The episodes tend to resolve spontaneously within two to five days but can recur multiple times over months to years. The true incidence of Mollaret’s syndrome remains unknown but is likely underreported [3].

CSF findings typically reveal lymphocytic pleocytosis, mildly elevated protein, and normal or slightly decreased glucose levels. Clinical features and cerebrospinal fluid profiles in recurrent HSV-2 meningitis remain consistent across large cohorts. Choi et al. found that most patients with recurrent HSV-2 meningitis presented with lymphocytic pleocytosis, normal glucose, and slightly elevated protein levels during symptomatic episodes [5]. These findings reinforce the reliability of CSF patterns and PCR for diagnosis, as seen in our case. PCR of CSF has emerged as the gold standard diagnostic tool for HSV-2 meningitis, enabling a specific diagnosis and sparing patients unnecessary antibiotics. Without PCR confirmation, many cases are incorrectly attributed to idiopathic viral meningitis or even bacterial meningitis [6].

Management of acute episodes involves high-dose antiviral therapy, usually with acyclovir or valacyclovir, and supportive care [7]. The role of long-term suppressive antiviral therapy remains controversial but may be considered in patients with frequent, debilitating recurrences [8]. The British National Formulary recommends initiating oral valacyclovir 500 mg once or twice daily as a suppressive option for recurrent HSV infections [8]. The duration of suppressive therapy is not typically lifelong; rather, it is usually trialed for several months to a year, with periodic reassessment of recurrence frequency, tolerability, and patient preference. Continuation or discontinuation should be individualized, as some patients experience reduced relapse rates over time

In addition to virologic considerations, the broader impact on patient well-being must not be overlooked. The relapsing nature of the illness can have a profound psychological toll. Patients often experience anticipatory anxiety, fear of recurrence, and frustration stemming from prolonged diagnostic uncertainty. Patel et al. reported that individuals with recurrent HSV-2 meningitis scored significantly lower in quality of life domains and often developed anxiety or depressive symptoms in response to the unpredictability of relapses [9]. Such outcomes emphasize the importance of involving psychiatric or psychological support services in long-term care planning.

Furthermore, a study by Tyring et al. demonstrated that education and anticipatory guidance provided during initial diagnosis were associated with improved coping and reduced health care utilization in patients with recurrent HSV-2 meningitis. As demonstrated in our case, multidisciplinary management, including early neurology involvement, patient education, and psychiatry liaison, can mitigate the psychosocial burden and empower patients to make informed decisions about suppressive treatment options.

This case underscores the need for heightened clinical suspicion of HSV-2 in patients with recurrent aseptic meningitis. Evaluation for underlying immunosuppression, including HIV infection, is also crucial. Our patient had negative HIV serology and no risk factors for acquired immunodeficiency, consistent with Mollaret’s meningitis as an isolated phenomenon rather than secondary to an immunocompromised statePrompt CSF PCR testing can help confirm the diagnosis, avoid unnecessary broad-spectrum antibiotics, and guide appropriate therapy. Education of patients about the benign nature and likelihood of recurrence is also crucial for reducing anxiety and inappropriate hospital admissions.

This case report has some limitations. First, long-term follow-up was not available, which restricts our ability to comment on recurrence risk after discharge or the eventual need for suppressive therapy. Second, the empirical use of IV chloramphenicol-though justified by the patient’s severe β-lactam allergy-represents an unusual treatment regimen compared to current standard practice, which may limit generalizability of the management approach. These factors should be taken into account when interpreting the findings.

## Conclusions

This case underscores the necessity of considering Mollaret’s meningitis in patients who present with recurrent episodes of aseptic meningitis. As shown here, PCR testing of the CSF for HSV-2 plays a critical role in establishing an accurate diagnosis and guiding appropriate therapy. Recognizing the self-limiting and benign nature of the disease is essential to avoid the overuse of unnecessary and potentially harmful antimicrobial therapies. Furthermore, patient education regarding the recurrent but non-life-threatening course of the condition can help reduce anxiety and improve quality of life. Long-term suppressive antiviral therapy may be considered for those with frequent or severe relapses, and such decisions should be individualized. This case highlights the importance of clinical vigilance, early and appropriate use of diagnostic tools, and patient-centered management in achieving optimal outcomes in Mollaret’s meningitis.
